# Weather Conditions and COVID-19 Incidence in a Cold Climate: A Time-Series Study in Finland

**DOI:** 10.3389/fpubh.2020.605128

**Published:** 2021-02-25

**Authors:** Behzad Heibati, Wenge Wang, Niilo R. I. Ryti, Francesca Dominici, Alan Ducatman, Zhijie Zhang, Jouni J. K. Jaakkola

**Affiliations:** ^1^Faculty of Medicine, Center for Environmental and Respiratory Health Research, University of Oulu, Oulu, Finland; ^2^Faculty of Medicine, Biocenter Oulu, University of Oulu, Oulu, Finland; ^3^Medical Research Center Oulu, Oulu University Hospital, University of Oulu, Oulu, Finland; ^4^Department of Epidemiology and Health Statistics, School of Public Health, Fudan University, Shanghai, China; ^5^Department of Biostatistics, T. H. Chan School of Public Health, Harvard University, Boston, MA, United States; ^6^West Virginia University School of Public Health, Morgantown, WV, United States; ^7^Finnish Meteorological Institute, Helsinki, Finland

**Keywords:** COVID-19, cold climate, weather, Finland, air pollution

## Abstract

**Background:** The current coronavirus disease 2019 (COVID-19) is spreading globally at an accelerated rate. There is some previous evidence that weather may influence the incidence of COVID-19 infection. We assessed the role of meteorological factors including temperature (T) and relative humidity (RH) considering the concentrations of two air pollutants, inhalable coarse particles (PM_10_) and nitrogen dioxide (NO_2_) in the incidence of COVID-19 infections in Finland, located in arctic-subarctic climatic zone.

**Methods:** We retrieved daily counts of COVID-19 in Finland from Jan 1 to May 31, 2020, nationwide and separately for all 21 hospital districts across the country. The meteorological and air quality data were from the monitoring stations nearest to the central district hospital. A quasi-Poisson generalized additional model (GAM) was fitted to estimate the associations between district-specific meteorological factors and the daily counts of COVID-19 during the study period. Sensitivity analyses were conducted to test the robustness of the results.

**Results:** The incidence rate of COVID-19 gradually increased until a peak around April 6 and then decreased. There were no associations between daily temperature and incidence rate of COVID-19. Daily average RH was negatively associated with daily incidence rate of COVID-19 in two hospital districts located inland. No such association was found nationwide.

**Conclusions:** Weather conditions, such as air temperature and relative humidity, were not related to the COVID-19 incidence during the first wave in the arctic and subarctic winter and spring. The inference is based on a relatively small number of cases and a restricted time period.

## Background

In Finland, the first case of COVID-19 was identified in the Helsinki Metropolitan Area on January 29, 2020. Since then, the SARS-CoV-2 pandemic has spread to other regions of the country via travelers from Helsinki or foreign countries. To supplement other major mechanisms promoting public safety during the pandemic, on March 12 the Finnish government declared the Emergency Powers Act, which was approved in the Parliament and came into effect on March 16. This Act enabled the Government to decide about several recommendations and orders aiming at controlling the SARS-CoV-2 pandemic. Among the actions, schools and day-care centers were closed, the province of Uusimaa, including the Helsinki metropolitan area, was isolated from the rest of the country and several restrictions and recommendations were made for unnecessary travel.

Several epidemiological studies in an early phase of the pandemic suggested that weather may influence the incidence of SARS-CoV-2 virus infection (1–4). The first results from Wuhan reported an association of COVID-19 mortality with diurnal temperature range and low humidity (2). In a study of 17 Chinese cities, COVID-19 incidence was inversely related to an increase in temperature and diurnal temperature range (4). Two studies conducted in subtropical cities of Brazil both provided evidence that an increase in temperature is related to a decrease in COVID-19 incidence (1, 3). This is consistent with previous evidence on the associations between weather and temperature *per se* for several other viral diseases, including SARS-CoV (5), *h. influenza* (6), and *rhino* viruses (7). A Chinese study based data from Wuhan and XiaoGan from Jan 26th to Feb 29th reported a positive correlation of COVID-19 incidence with daily air quality index, PM_2.5_, NO_2_ concentrations, but a negative correlation with temperature (8). There is also recent evidence from a US nationwide study that long-term exposure to air pollutants is related to an increased risk of COVID-19 infection at a community level, indicating that air pollution may increase susceptibility to COVID-19 infections (8, 9). Environmental factors may influence function of the virus itself, but it is also likely that these factors predispose individuals to infection by pathophysiological and immunological responses to the environmental challenges (6). However, the independent effect of environmental factors on the incidence of COVID-19 in Finland has not been studied.

Based on previous evidence on COVID-19 (1–4) and other viral pathogens (5–7) and the substantial evidence on the influence of cold weather to human health and immunology (10, 11), we hypothesized that cold temperature and low relative humidity increase the incidence of COVID-19 infection in the cold climate. We tested this hypothesis by assessing the relations between meteorological factors, including daily ambient temperature (T) and relative humidity (RH), and daily counts of COVID-19 cases in Finland adjusting for air pollutants (PM_10_ and NO_2_) during the first 5 months of the pandemic.

## Methods

### Study Area

**Figure 1:**
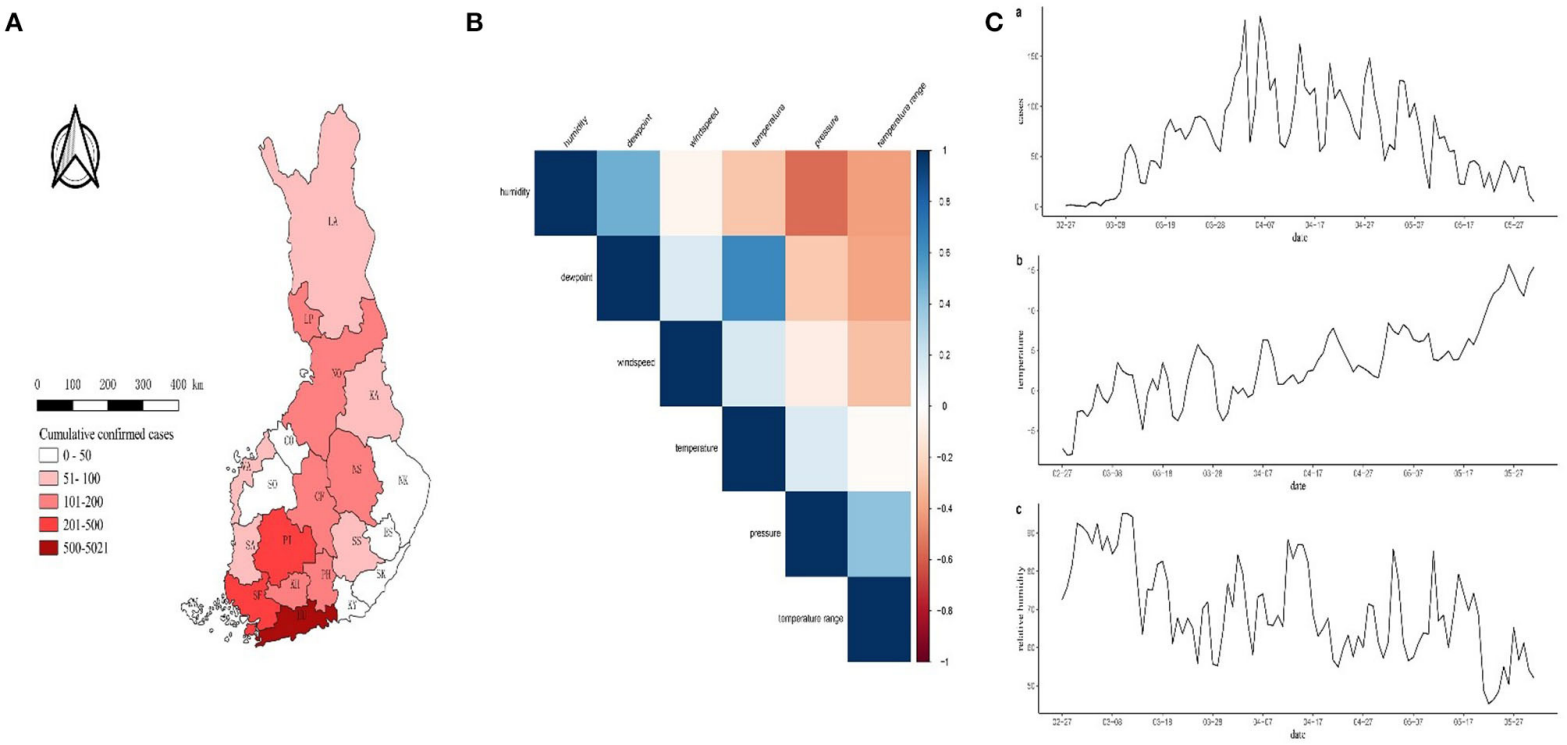
**(A)** Number of confirmed COVID-19 cases in the hospital districts. **(B)** Plot of correlation coefficient matrix. Darker blue colors indicate higher correlation between the two variables. **(C)** Time series plot of (a) COVID-19 cases, (b) temperature (T), and (c) relative humidity (RH). The hospital districts are Helsinki and Uusimaa (HU), Soutwest Finland (SF), Pirkanmaa (PI), Länsi-Pohja (LP), Northern Ostrobothnia (NO), North Savo (NS), Central Finland (CF), Kanta-Häme (KH), Päijät-Häme (PH), Lapland (LA), Kainuu (KA), Vaasa (VA), Satakunta (SA), South Savo (SS), South Ostrobothnia (SO), Kymenlaakso (KY), North Karelia (NK), South Karelia (SK), Central Ostrobothnia (CO), East Savo (ES) and Ahvenanmaa (AL).

The study area comprised the whole Finland, located between latitudes 60° and 70°N, and longitudes 20° and 32°E, with a population of 5.5 million (Figure 1A). The Capital city of Helsinki and the surrounding cities comprise the Helsinki Metropolitan area with altogether 1.1 million inhabitants. Finland is located between the Baltic Sea and the Eurasian continent and has characteristics of both maritime and continental climates. The mean annual temperature is 6.6°C and the mean annual and monthly precipitation in July are 655 and 63 mm, respectively (Finnish Meteorological Institute; http://ilmatieteenlaitos.fi/).

### COVID-19 Data

The National Institute for Health and Welfare of Finland (https://thl.fi/en/web/thlfi-en) maintains the registry of infectious diseases in Finland, and the law requires that health care personnel report all cases of selected infectious diseases including COVID-19. Thus, selection bias by underreporting is minimal. We retrieved the daily count data of the confirmed coronavirus cases (COVID-19) from January 1 to May 31, 2020 from the official website of National Institute for Health and Welfare of Finland (https://experience.arcgis.com/experience/92e9bb33fac744c9a084381fc35aa3c7).

### Meteorological and Air Quality Data

The daily contemporaneous meteorological data, including daily average temperature (T, °C), average relative humidity (RH, %), dew point (°C), wind speed (m/s) and pressure (KPa) was retrieved from the Finnish Meteorological Institute (https://en.ilmatieteenlaitos.fi/). These data are based on continuous measurement of the weather stations across the country. For each hospital district, we calculated an average value from the stations closest to the provincial hospital. We calculated daily temperature range as a difference between the daily maximum and daily minimum temperature. Data on daily concentrations of air pollutants, including NO_2_ (μg/m^3^) and PM_10_ (μg/m^3^), were also retrieved from the Finnish Meteorological Institute and were treated as potential confounders in the sensitivity analyses.

### Statistical Analysis

Time-series methods were used to assess the associations between daily meteorological factors and daily count of COVID-19 cases. A standard two-stage approach was applied to obtain the region-specific and nationwide incidence rate ratios (IRRs) as measures of effect. In the first stage, a quasi-Poisson generalized additional model (GAM) was fitted to estimate the relations between region-specific meteorological factors and COVID-19 incidence rate (IR). Considering the reliability of the models, only regions with >100 cases (*N* = 9) were included in the analysis and 12 other districts were excluded from the study. The nine regions produced 93% of the COVID-19 cases. Spearman correlation coefficient matrix was calculated for meteorological factors in each region, and the correlation matrices were pooled by averaging the region-specific correlation coefficients. To avoid multicollinearity, the threshold of correlation coefficient was set as 0.6. We applied the backward elimination algorithm for selection of variables to the final model in each district. Wald test was used for testing statistical significance. The effects of meteorological factors were expressed with a 14-day exponential moving average (EMA) (12). To control the short-term temporal trend, the natural splines of time with 2 degrees of freedom was applied. The model is given by:

$$\begin{array}{l} E\left(y_{t}\right)\;=\;\mu_{t}\\ log\;\mu_{t}\;=\;\beta_{0}\;+\;\beta_{1}\times matem\;p_{t}\;+\;\beta_{2}\times mahm\;d_{t} \end{array}$$

Where, *y*_*t*_ is the daily count of COVID-19 at day *t*, μ_*t*_ is the expected value of daily count at day *t*, β_0_ is the intercept, β_1_ and β_2_ denote the effect of moving average of temperature and relative humidity, and β_3_ and β_4_ are the regression coefficients of natural splines of time with two degrees of freedom.

In the second stage, a meta-regression model with random effects was used to obtain national average effect estimate between COVID-19 and meteorological factors. *I*^2^ statistics and Cochran Q test were used to quantify inter-regional spatial heterogeneity. To estimate the overall relationship of the association between meteorological factors and COVID-19, exposure-response curves were plotted using the GAM with natural spline's knot setting at its median (*df* = 2).

### Sensitivity Analyses

Sensitivity analyses were performed by modifying the parameter of EMA from 14 days to 10 and 12 days, respectively, and including the two air pollutants (PM_10_, NO_2_) into the above model as potential confounders to assess their possible influence on the associations between meteorological factors and COVID-19 incidence. The R4.0.1 software (R Foundation for Statistical Computing, Vienna, Austria) was used to perform all analyses. ArcGIS10.1 software (Environmental Systems Research Institute Inc, Redlands, CA, USA) was used to draw the geospatial map.

## Results

### Characteristics of the COVID-19 Pandemic in Finland

A total of 6,831 cases of COVID-19 were confirmed in Finland during the study period January 1 to May 31, 2020. The cumulative number of confirmed cases appears to have a gradually decreasing trend from south to north (Figure 1A).

### Region-Specific Analysis

Regions with >100 cases were Helsinki and Uusimaa (HU), Southwest Finland (SF), Pirkanmaa (PI), Länsi-Pohja (LP), Northern Ostrobothnia (NO), North Savo (NS), Central Finland (CF), Kanta-Häme (KH), and Päijät-Häme (PH) districts in our analysis shown in Figure 1. Dewpoint was excluded from the correlation coefficient matrix (Figure 1B) and temperature range, wind speed and pressure were excluded from the univariate GAM model. Only average temperature and relative humidity were selected in the final model, which ranged from −8.03 to 15.70 (°C), and from 45.31 to 95.16%, respectively (Figure 1C). The COVID-19 incidence rate gradually increased until a peak around April 6 and then decreased to May 31 (Figure 1C,a), while temperature had a visibly upward trend (Figure 1C,b) and relative humidity had a downward trend in the same time period (Figure 1C,c).

**Figure 2:**
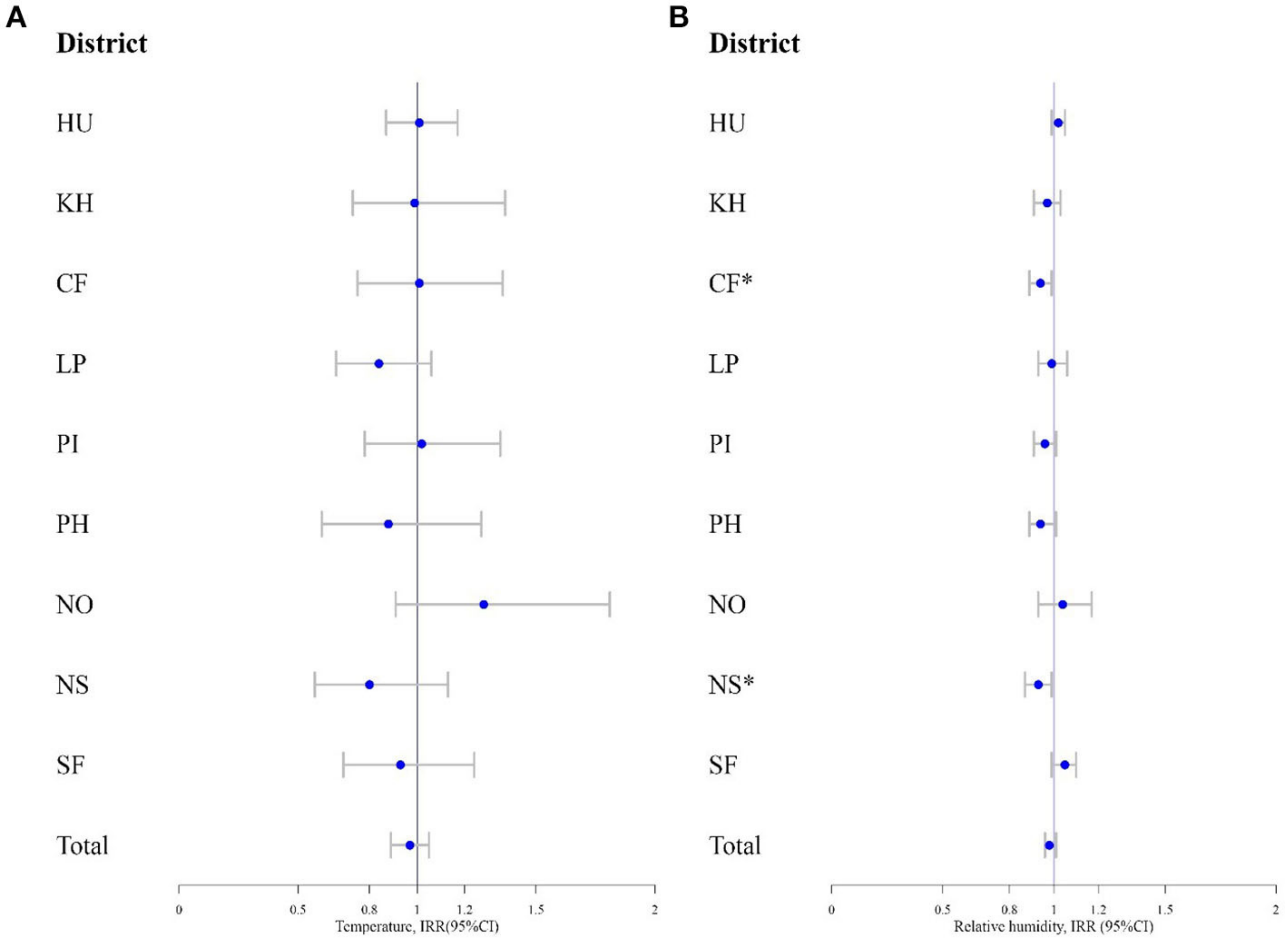
**(A)** The incidence rate ratio (IRR) and 95% confidence interval (95% CI) for COVID-19 by temperature and **(B)** relative humidity. * indicates statistical significance.

Overall, there was no association between temperature or relative humidity and nationwide incidence rate of COVID-19, although both showed a tendency of negative association (Table 1). However, there was a spatial heterogeneity in the associations between relative humidity and COVID-19 across regions (*I*^2^ 65.11%). In Central Finland (CF) and North Savo (NS), relative humidity was negatively correlated with COVID-19, but in other regions it was not. Temperature was not associated with COVID-19 in any of the regions (Figure 2).

**Table 1:** Nationwide associations between temperature and relative humidity and COVID-19 incidence in Finland.

**Variable**	**Estimate (95% CI)**	***I*^**2**^ (%)**	**Cochran Q test**	
			**Statistics**	***P-value***
Temperature	−0.03 (−0.11, 0.05)	<0.01	6.14	0.63
Relative humidity	−0.02 (−0.04, 0.0001)	65.11	22.93	<0.01

### Exposure-Response Curves

**Figure 3:**
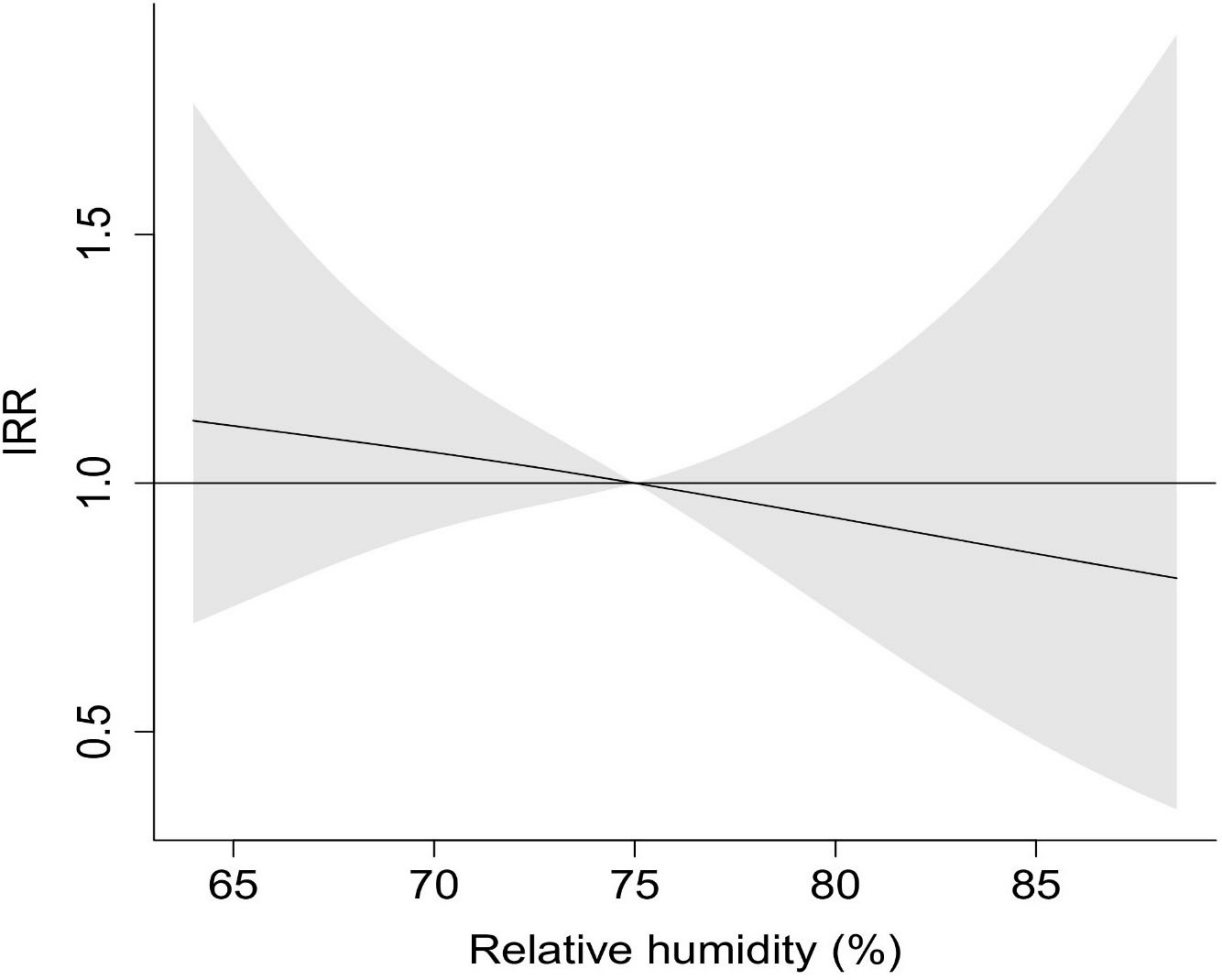
Exposure-response curve of IRR for COVID-19 by relative humidity. Ninety-five percentage confidence interval is shown shaded.

The exposure-response curves depicted the slightly decreasing linear tendency for the association of relative humidity with COVID-19 incidence (Figure 3).

### Sensitivity Analysis

The sensitivity analysis indicates the results are robust under the situations of changing the EMA parameter or including the air pollutants as potential confounders (Table 2).

**Table 2:** Sensitivity analyses on the associations between temperature and relative humidity and COVID-19 incidence in Finland [Estimate (95% CI)].

**Variable**	**EMA (*****df*****)**		**Air pollutants**
	**10**	**12**	**(PM_10_ + NO_2_)**
Temperature	−0.02 (−0.08, 0.04)	−0.02 (−0.1, 0.06)	−0.04 (−0.13, 0.05)
Relative humidity	−0.01 (−0.03, 0.01)	−0.01 (−0.03, 0.01)	−0.01 (−0.05, 0.02)

## Discussion

### Main Findings

On the basis of previous knowledge about weather and COVID-19 incidence (1–4) and several other viral diseases, including SARS-CoV (5), *h. influenza* (6), and *rhino* viruses (7), we tested the hypothesis that low temperature and low relative humidity increase the incidence of COVID-19. We evaluated the relations between weather conditions and the daily number of COVID-19 cases in Finland during the first 5 months of the pandemic. In the nationwide analysis, temperature and relative humidity were not associated with the incidence of COVID-19. However, in two hospital districts located inland there was a negative association between relative humidity and COVID-19 incidence i.e., consistently with our hypothesis, the incidence rate of COVID-19 was greater in the dry air.

### Validity of the Results

We used data from the meteorological stations located in the same city with the provincial hospital. This approach assumes that the spatial distribution of exposure is homogeneous. This assumption is reasonable for temperature and relative humidity but results in measurement error in air pollution concentrations. Exposure assessment was made at population-level, which means that there was no information on individual-level exposure.

The infectious disease law required reporting of all COVID-19 cases to the National Institute of Health and Welfare of Finland. The identification of severe cases of COVID-19 was likely to be complete, but the percentage of non-diagnosed mild cases is unknown. Further, the percentage may have changed over time. The diagnostic practice was changing over the course of the pandemic producing uncertainty to the incidence rate estimates. However, misclassification or underdiagnosis of COVID-19 was not likely to be related to prevailing weather and thus any systematic error was not likely. There is a possibility that some cases were first identified outside their own hospital district. This would cause bias if their exposure was based on the conditions in their home district. Due to common public patient information databases, the information could be in most cases seen both hospital districts, which would have reduced the potential bias.

The population producing the COVID-19 cases remained relatively constant. Air pollution was the most obvious potential confounder, because weather, especially temperature is for several reasons associated with air pollution concentrations. We were able to adjust for PM_10_ and NO_2_, but as stated before the concentrations at the monitoring stations may not be representative of the whole hospital districts. However, the concentrations at the monitoring stations give reasonable estimates of the relative levels of air pollution over time. Inclusion of PM_10_ and NO_2_ did not influence the associations between weather parameters and COVID-19 incidence.

We applied a statistical approach, a quasi-Poisson generalized additional model, commonly used in chronic disease epidemiology where the assumption of the independence of individual observations is reasonable. The dynamics of COVID-19 pandemic is new and unknown which is a source of uncertainty when assessing the effects of weather on disease incidence. For example, the emergence of the COVID-19 pandemic took place from early winter to late spring during which there is a strong time-trend of both temperature and relative humidity. Never-the-less, we think it was important to make a fast attempt to model the associations between the main weather parameters and the incidence rate of COVID-19 regionally and nationwide. In reality, the incidence rate of COVID-19 declined fast after the intensive intervention on March 16, which definitely played a role in the reduction. This intervention may have masked the influence of increasing ambient temperature in the course of changing season from winter to summer. Further, we were not able to take into account any population movements related to tourism or immigration, which were likely to influence the COVID-19 incidence.

### Synthesis With Existing Knowledge

We identified several previous studies which had assessed the role of weather in the COVID-19 incidence (1–4, 12–23), but only few studies were conducted in a cold climate with large temperature variations. A synthesis of the present and previous findings faces thus several challenges. The studies were conducted in different climatic zones, the relations between weather parameters and COVID-19 incidence rate were estimated in different ranges of temperature and relative or absolute humidity, and there were substantial differences in the statistical modeling approaches.

One of the studies by Bashir et al. (15) was conducted in New York City with a climate and temperature range closest to Finland, two Chinese studies covered several climatic zones (16, 18), the Brazilian study by Prata et al. (3) included all the 27 state capitals all in subtropical and tropical climates and the study in New South Wales, Australia by Ward et al. was conducted in subtropical climate (12). Another study by Pramanik et al. was conducted in the Russian climatic regions with a similar range of climate and temperature as in Finland, including a total of 101 primarily selected cities classified into two climatic regions (79 cities in the humid continental and 22 cities in the sub-arctic climate) (19).

We did not find any overall or regional association between daily temperature and incidence rate of COVID-19 during the study period with a temperature range from −8.0 to 15.7°C. Bashir et al. (15) reported significant correlations between daily average and minimum temperatures and the daily count of COVID-19 in New York City. The range of temperature was from −1.8 to 15.7°C, which partially overlaps with the temperature range in the present study. The statistical analysis was based on calculation of Kendall and Spearman correlation between daily air quality parameters and counts of COVID-19 cases during March 1 – April 12, 2020. In the Russian study, the temperature seasonality (29.2 ± 0.9%) had the strongest effect on incidence of COVID-19 in the humid continental region. The authors reported that the diurnal temperature range (26.8 ± 0.4%) and temperature seasonality (14.6 ± 0.8%) had the greatest contribution for incidence in the sub-arctic region (19). On the other hand, the effects of diurnal temperature range, wind speed, and relative humidity on the intensity of the COVID-19 incidence were observed in the sub-arctic region. The temperature was relatively low (<2°C) which overlaps with the temperature range in our study. Pramanik et al. (19) reported that COVID-19 risk was lower in the temperate and subtropical regions when the temperature remains <10°C.

Berumen et al. analyzed the effects of temperature and humidity on the doubling time of COVID-19 cases in 67 countries grouped by the climate zone (20). This study suggested that the behaviors of the growth curve and doubling time in the first stage of the epidemic were related to the ambient temperature but the magnitude of this effect was different between countries located in temperate and tropical/subtropical areas (20). Two studies in tropical climate provided controversial results of the association between temperature and COVID-19 risk. In the Brazilian study, there was a 4.9% decrease in COVID-19 risk per 1°C increase in temperature ranging from 16.8 and 27.4°C, i.e., in a different range compared with that in Finland (3). In the study conducted in New South Wales, Australia there was no association between temperature and COVID-19 in a subtropical climate with a temperature ranging from 16 to 24°C (9 a.m.) and 16 to 34°C (3 p.m.) (12). In the Chinese study of 122 cities in different climatic zones, a non-linear association was reported between temperature and COVID-19 incidence (21). The incidence rate of COVID-19 increased by 4.9% per 1°C increase up to −3°C, but there was no association in warmer temperature range. In the other Chinese study of 80,981 COVID-19 cases in 31 provinces with 344 cities, the incidence rate of COVID-19 decreased by increase in temperature in the range of −22 to 26°C (22). The association between temperature and COVID-19 incidence appears to be non-linear with the highest risk in temperatures around zero. Although in the Finnish nationwide analysis there was no association between relative humidity and COVID-19, there was evidence of a negative association in two inland provinces. The range of RH was from 45.31 to 95.16%. Consistently with this observation, the New York study reported a negative association between relative humidity and COVID-19 incidence (15). Interestingly, a negative association between relative humidity and COVID-19 incidence was reported also in the subtropical climate of New South Wales, with a 6.11% risk increase by 1% reduction in relative humidity (12). The large Chinese study of 31 provinces in different climate zone found no association between relative humidity and COVID-19 incidence (23).

In summary, there seem to be differences in COVID-19 transmission between different climate zones (22), and these differences could be partially explained by weather. However, there are several population-level alternative explanations for the observed difference in COVID-19 transmission between geographical regions, including climate zones. Potential determinants are population density, age distribution, levels and differences in socioeconomic conditions, and any cultural and behavioral differences that could modify the actual exposures to weather conditions, such as differences in housing stock, heating infrastructures, and risk perception. It is also too early to elaborate the role of seasonality itself in this context, as not all seasons have been experienced in the COVID-19 pandemic yet. The associations between weather and COVID-19 transmission require further research. Our own results provide some preliminary evidence that low relative humidity may play a role in COVID-19 transmission.

## Conclusions

This nationwide time-series analysis of the Finnish COVID-19 cases during the early pandemic months did not provide evidence that ambient air temperature and relative humidity affected the COVID-19 incidence in the arctic and subarctic winter and spring. We provide suggestive evidence that dry air may increase the incidence of COVID-19. The inference is based on a relatively small number of cases and a restricted time period covering the first wave of the pandemic in Finland. Most of the previous studies were conducted in very different climates and ranges of temperature and relative humidity. However, the evidence of the role of temperature and relative humidity is controversial even in studies from similar climatic conditions. A non-linear association between temperature and COVID-19 risk could partly explain the controversial observations, whereas the complex dynamics of COVD-19 pandemic and radical social interventions may complicate the inference. Further studies are needed to elaborate on the complex associations between weather and COVID-19 in different climates and seasons. The emerging second wave of the pandemic in Finland will offer an opportunity for further assessment of these relations.

## Data Availability Statement

The raw data supporting the conclusions of this article will be made available by the authors, without undue reservation.

## Ethics Statement

This study was based on anonymous registry data.

## Author Contributions

BH: data collection, writing—original draft, writing—review and editing. WW: conceptualization, methodology, and formal analysis. NR: writing—review and editing. AD and FD: review and editing. ZZ: review and editing and supervision. JJ: supervision, writing—review and editing. All authors contributed to the article and approved the submitted version.

## Conflict of Interest

The authors declare that the research was conducted in the absence of any commercial or financial relationships that could be construed as a potential conflict of interest.
