# Supplementary material for: Weather Conditions and COVID-19 Incidence in a Cold Climate: A Time-Series Study in Finland
Source: Front Public Health. 2021 Feb 25;8:605128. doi: 10.3389/fpubh.2020.605128 (PMC7946816; doi:10.3389/fpubh.2020.605128)
Supplement: Supplementary file 1 [file Data_Sheet_1.pdf]

APPENDIX TABLE 1. Correlation coefficient matrix for the meteorological factors.

Variables	Humidity	Dewpoint	Windspeed	Temperature	Pressure	Temperature range
**Humidity**	1	0.49	-0.06	-0.27	-0.56	-0.41
**Dewpoint**	/	1	0.15	0.64	-0.26	-0.39
**Windspeed**	/	/	1	0.16	-0.09	-0.29
**Temperature**	/	/	/	1	0.16	-0.03
**Pressure**	/	/	/	/	1	0.41
**Temperature range**	/	/	/	/	/	1
